# Pediatric obesity and the risk of multiple sclerosis: a nationwide prospective cohort study

**DOI:** 10.1038/s41366-025-01727-3

**Published:** 2025-01-30

**Authors:** Emilia Hagman, Resthie R. Putri, Pernilla Danielsson, Claude Marcus

**Affiliations:** Division of Pediatrics, Department of Clinical Science, Intervention and Technology, Karolinska Institutet, S-141 57 Huddinge, Sweden

**Keywords:** Autoimmune diseases, Risk factors, Epidemiology

## Abstract

**Background:**

Emerging evidence implies a link between high pediatric body mass index (BMI) and an increased risk of multiple sclerosis (MS). However, previous research suggests this association is only present for adolescent obesity and not childhood obesity. The present study aimed to assess the association between pediatric obesity and risk of developing MS, and to investigate if degree of obesity and age at obesity treatment initiation affects the risk. In a subgroup, response to obesity treatment on MS risk was assessed.

**Methods:**

In this cohort study, patients aged 2–19 years from the Swedish Childhood Obesity Treatment Register (BORIS), and matched individuals from the general population were followed prospectively. MS was identified through the National Patient Register. Hazard ratios (HR) adjusted for parental MS were calculated.

**Results:**

The study included 21,652 individuals with pediatric obesity and 102,187 general population comparators. The median age at follow-up was 21 (Q1, Q3 18, 25) years. The adjusted HR (95% CI) for developing MS in the pediatric obesity cohort was 2.28 (1.45–3.58). In stratified analyses, obesity class I was not associated with MS, HR = 1.34 (0.64–2.81), while the association between obesity class II and MS was strengthened, HR = 3.42 (1.89–6.19). MS was associated with both childhood obesity, HR = 3.16 (1.12–8.87), and adolescent obesity, HR = 2.12 (1.28–3.51). A decrease in BMI SDS was not associated with lower likelihood of MS, HR = 1.09 (0.92–1.29) per 0.25 BMI SDS unit decrease.

**Conclusions:**

Both childhood and adolescent obesity are associated with an increased risk of MS. Moreover, a dose-response relationship between the degree of obesity and the risk of future MS was indicated, while response to pediatric obesity treatment did not affect the association, highlighting the importance of preventing high degree of obesity early in life.

## Introduction

Children and adolescents with obesity face a plethora of complications, including comorbidities, psychosocial challenges, and even premature mortality [1–4]. Emerging evidence implies a link between high body mass index (BMI) in childhood or adolescence and an increased risk of multiple sclerosis (MS) [5, 6]. MS is a chronic inflammatory condition characterized by demyelination, stemming from an autoimmune response targeting myelin. MS typically manifests between 15 and 55 years of age, affecting women two to three times more often than men [7]. High BMI in young years is estimated to contribute to more than 10% of MS cases [8]. Yet, most studies evaluating the association between pediatric obesity and risk for MS are relatively small cross-sectional [9], have a retrospective design with self-reported weight data [10–12], have used solely genetic correlations [13, 14], or use pediatric weight data before the obesity epidemic [5]. In addition, previous research suggest that the association only is present for adolescent obesity and not childhood obesity [15–17]. By the age of 20, individuals with a BMI exceeding 27 kg/m² have been found to have twice the risk of developing MS compared to those with a BMI of 18.5–21 kg/m² [6]. Further, there is lacking evidence on whether degree of obesity affects the risk of developing MS.

Therefore, we aimed to prospectively evaluate the risk of developing MS in a large cohort of pediatric patients with obesity and general population comparators, and to investigate if degree of obesity and age at obesity treatment initiation affects the association. Additionally, we aimed to evaluate whether response to obesity treatment affected the risk of MS.

## Methods

### Study population

**Fig. 1:**
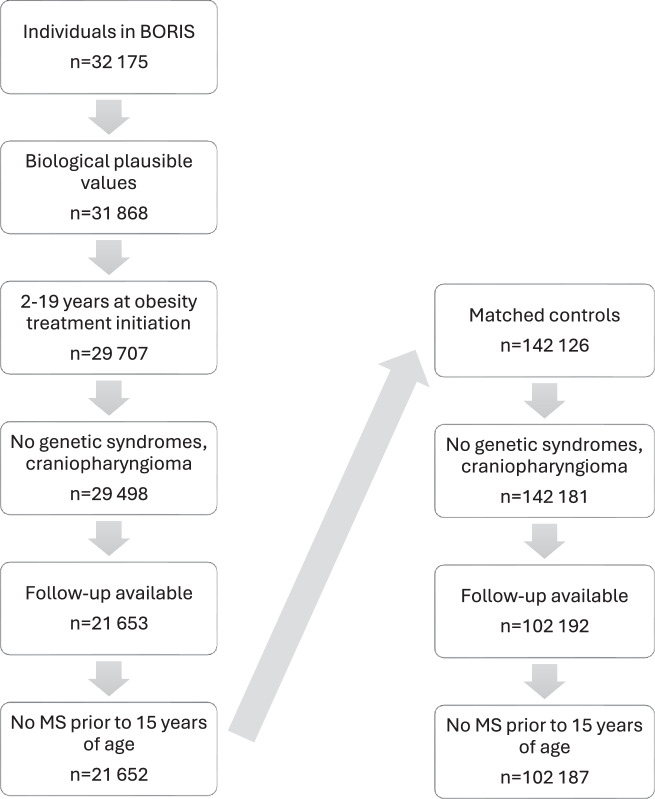
Flowchart of inclusion. Biological plausible values include BMI 15–90 kg/m^2^, and height Z-score between −5.0 and 5.5. No available follow-up includes individuals under 15 years of age, deceases or emigrated at start of follow-up.

In this dynamic cohort study, we included patients aged 2–19 years with obesity [18] enrolled in the Swedish Childhood Obesity Treatment Register (BORIS) between January 1997 and December 2020. BORIS is a national prospective register where children and adolescents receiving obesity treatment in Sweden are recorded [19]. The treatment primarily involves behavioral lifestyle modification. During the obesity treatment period of the current study, the new generation anti-obesity medications was not approved for adolescents in Sweden. Therefore, no patients received pharmacological treatment for obesity. Swedish law and regulations currently allow clinicians to register their patients in BORIS unless the patient chooses to opt-out. For each patient in BORIS, up to five individuals from the general population were contemporary matched on sex, year of birth, and residential area from the Total Population Register, held by Statistics Sweden [20]. Exclusion criteria were secondary obesity (craniopharyngioma), genetic syndromes (e.g. Mb Down, Prader Willi, and Laurence Moon Biedl, see Supplementary Table 1 for full list), and emigrated, deceased, or MS diagnosis before 15 years of age or before obesity treatment initiation. Flowchart of inclusion is presented in Fig. 1. Individuals were followed from obesity treatment initiation, or from 15 years of age if obesity treatment was initiated earlier, until MS diagnosis, death, emigration, or August 2023, whichever came first. Date of death was retrieved from the Cause of Death Register and date of emigration was provided by Statistics Sweden. The study was approved by the regional ethical review board in Stockholm (No. 2016/ 922-31/1 and amendment 2020-02707).

### Variables and definitions

For all included individuals, MS was identified through the Swedish National Patient Register, held by the National Board of Health and Welfare, using International Statistical Classification of Diseases 10^th^ revision code G35. The certainty of MS diagnoses in the National Patient Register is very high (>90%) [21].

Since approximately 30% of MS risk is genetic [22], data of biological parents to individuals in the obesity cohort and the general population comparators were identified and retrieved from Statistics Sweden. Missing data of biological parents, e.g. internationally adopted children, was present in 0.56% in the obesity cohort and 0.92% among the general population comparators. Heredity for MS was deemed if any of the biological parents had MS identified in the National Patient Register.

Age was calculated from the date of birth, which, along with sex, was retrieved from Statistics Sweden. Age at pediatric obesity treatment initiation was categorized into two groups; childhood (2–9.9 years) and adolescence (10–19 years) [23]. Degree of obesity was categorized as class I and class II obesity according to International Obesity Task Force criteria [18]. Obesity treatment response was assessed as body mass index standard deviation score (BMI SDS) [18] reduction (per 0.25 unit).

### Statistical analyses

Descriptive characteristics are presented with proportions, mean and standard deviation (SD), or median and quintile 1 (Q1) and quintile 3 (Q3). Unadjusted incidence rate and associated 95% confidence interval (CI) as well as Cox proportional hazard ratio (HR) and 95% CI adjusted for heredity was calculated with attained age as time scale. Proportional hazard assumption was confirmed. Analyses were further stratified for sex, degree of obesity at obesity treatment initiation (obesity class I and obesity class II), and age at obesity treatment initiation. Given the higher validity of repeated MS diagnoses in the National Patient Register [21], the HR was also estimated for repeated diagnosis of MS in sensitivity analyses, requiring at least one diagnosis to be the main diagnosis. Among individuals in the pediatric obesity cohort, treatment response was assessed among those with at least one year (365 days) of treatment data. Covariate in the sub-analysis included parental MS. Missing data was handled using listwise deletion. All analyses were performed in SAS statistical software (version 9.4, Cary, NC, USA).

## Results

We included 21,652 individuals from the pediatric obesity cohort and 102,187 general population comparators. In the obesity cohort, 54% were males, the median (Q1, Q3) age at obesity treatment initiation was 11.5 (9.0, 13.9) years, and the distribution of obesity class I and class II were 58% and 42% respectively. The median age at start of follow-up was 15.0 (15.0, 15.0) years and follow-up time was 5.6 (3.0, 9.6) years, corresponding to 20.6 (18.0, 24.6) years of age. Parental MS was present in 0.99% in the obesity cohort and 0.69% in the general population comparators, *p* < 0.0001.

Over 144,970 person-years, 0.13% (*n* = 28) developed MS in the pediatric obesity cohort, compared to 0.06% (*n* = 58) over 698,513 person-years among the general population comparators, *p* = 0.0002. The mean (SD) age of MS diagnosis was comparable between the groups: 23.4 (5.0) years in the obesity cohort versus 22.8 (5.9) years in the general population comparators, *p* = 0.64. The distribution of sexes was similar across both groups: In the obesity cohort, females accounted for 64.3% (*n* = 18) of MS cases, compared to 65.5% (*n* = 38) the general population comparators, *p* = 0.91.

**Fig. 2:**
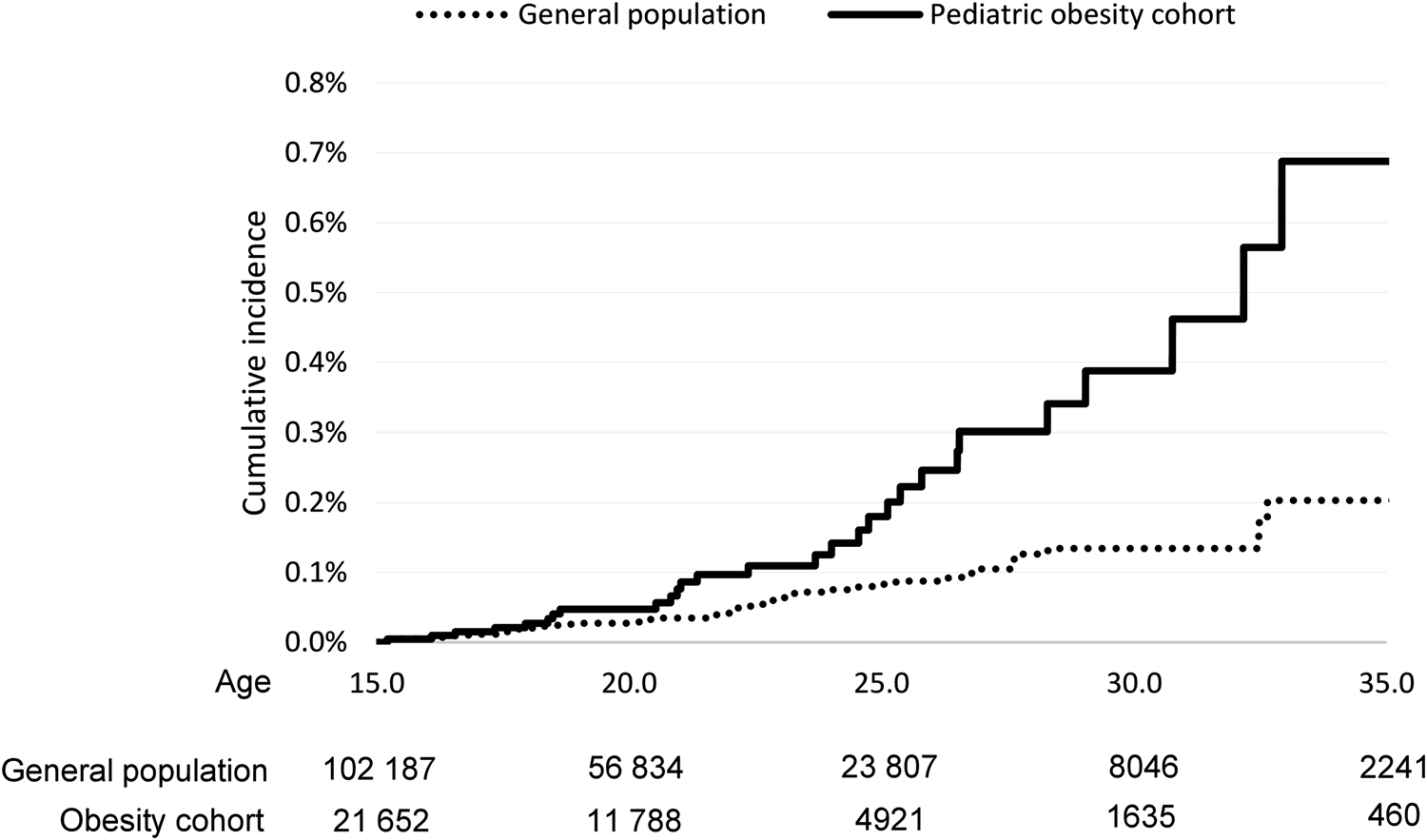
Unadjusted cumulative incidence of multiple sclerosis from 15 to 35 years of age. Number of individuals at each age are displayed per group below the graph.

The unadjusted incidence rate of MS per 100,000 person years was 19.3 (95% CI: 13.3–28.0) in the obesity cohort and 8.3 (6.4–10.7) among the general population comparators, *p* = 0.0002. By the age of 30, the estimated cumulative incidence of MS was 0.39% in the obesity cohort versus 0.13% in general population comparators, as illustrated in Fig. 2. Cox proportional hazard regression, adjusted for MS heredity, revealed that the risk of developing MS was more than twice in the pediatric obesity cohort compared to the general population, HR = 2.28 (95% CI 1.45–3.58), *p* = 0.0003, Table 1.

**Table 1:** Hazard ratios (95% CI) for multiple sclerosis.

	Unadjusted	Mutually adjusted
Pediatric obesity cohort vs. general population comparators	2.33 (1.48–3.65)	2.28 (1.45 – 3.58)
Parental MS (yes vs. no)	8.85 (3.86–20.31)	8.43 (3.67 – 19.35)

**Fig. 3:**
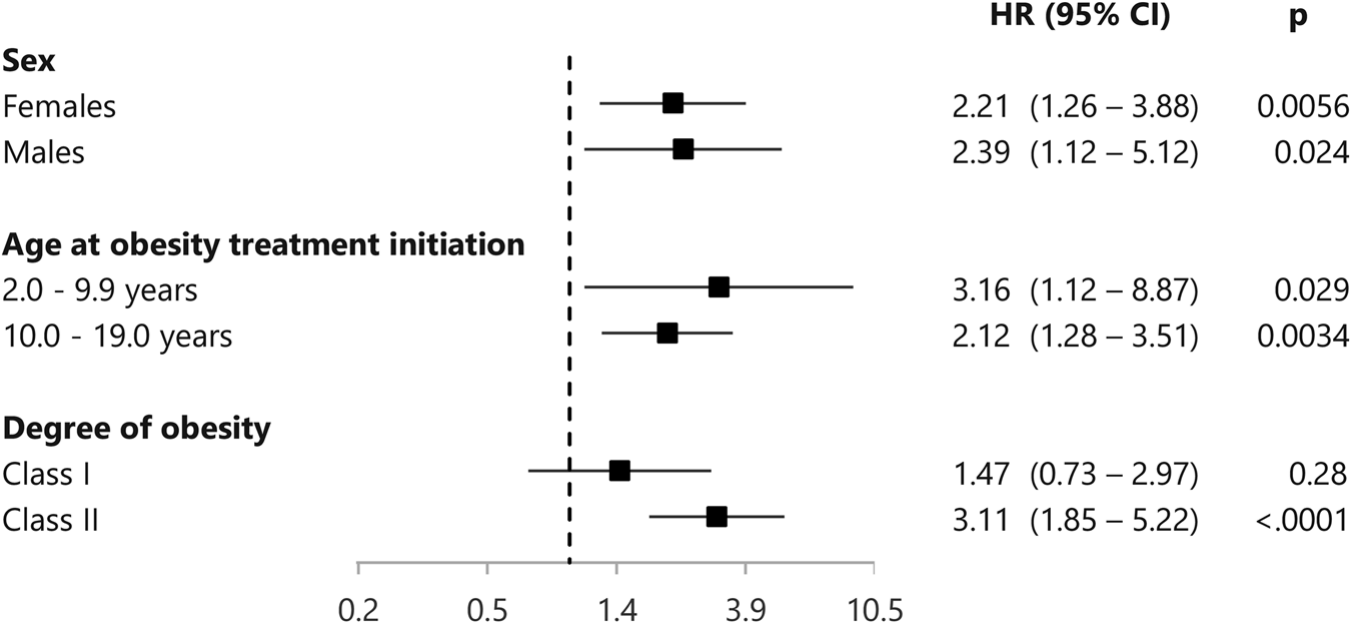
Hazard ratios of pediatric obesity cohort vs. general population comparators for multiple sclerosis stratified for sex, age and degree of obesity at obesity treatment initiation. Analyses were adjusted for parental MS. Dashed line- represents HR = 1.0.

There were no interactions between the exposure (pediatric obesity cohort or general population comparators) and sex (*p* = 0.90), age at treatment initiation (*p* = 0.66) on the risk of MS. In stratified analyses, obesity class I was not associated with MS, HR = 1.47 (0.73–2.97), while the association between obesity class II and MS was strengthened, HR = 3.11 (1.85–5.22). Stratified analyses are provided in Fig. 3.

In sensitivity analyses, repeated MS diagnosis was assessed. Seven individuals (three in the obesity cohort and four among general population comparators) with single diagnosis of MS were excluded. Of them, two had an MS diagnosis within two months before the end of follow-up. The association between pediatric obesity and risk of MS remained, adjusted HR = 2.18 (1.36–3.51), *p* = 0.0013.

Among individuals in the pediatric obesity cohort, data of at least one year of treatment was available in 64.6% (*n* = 13,991), of whom 0.13% (*n* = 18) was diagnosed with MS, excluding one patient diagnosed with MS prior to end of obesity treatment. The median (Q1, Q3) treatment duration was 3.1 (1.9, 5.0) years with a treatment response of −0.14 (−0.51, 0.13) BMI SDS units. The median time to MS diagnosis after last registered visit to pediatric obesity treatment was 8.1 (5.7, 10.8) years. In the sub-analysis, adjusted for parental MS, a decrease in BMI SDS was not associated with higher likelihood of MS, HR = 1.09 (0.92–1.29) per 0.25 BMI SDS unit decrease. Obesity treatment duration did not affect the outcome. Of those who developed MS, 17% obtained obesity remission, 28% had obesity class I and 55% had obesity class II the last registered visit to obesity treatment.

## Discussion

The current prospective nationwide cohort study shows that obesity prior to 10 years of age more than doubles the risk of MS regardless of MS heredity, compared to general population comparators. This study also confirms that obesity in adolescence increases the risk for MS. Additionally, this study suggests a dose-response relationship between degree of obesity and the risk for MS.

In the present study, the estimated risk of obesity on MS was more than double, which is in line with previous studies in adults [6, 15, 17] and studies with retrospective design with self-reported or estimated weight data from pediatric years [10–12]. The proportion of males and females that were diagnosed with MS were similar, which was mirrored in the hazard ratio estimates, indicating that obesity affects MS equally in males and females.

This study found that, compared to the general population, class I obesity did not significantly increase the risk for MS, while class II obesity showed a pronounced risk, indicating a dose-response relationship. The underlying mechanism may be attributed to obesity’s role in inducing a persistent, low-grade inflammatory state characterized by elevated levels of proinflammatory cytokines [2]. Type of diet and microbiota may also contribute both to obesity and MS [7]. A study in mice suggest that high-fat diet induced obesity causes a pro-inflammatory micro-environment which promotes CNS inflammation through proliferation and activation of encephalitogenic T cells into the CNS [24]. A cross-sectional study in humans suggest that leptin, typically high in individuals with obesity, promotes autoreactive T-cell proliferation and proinflammatory cytokine secretion [10]. Further, obesity-induced gut microbiota dysregulation has been shown to shift the balance between proinflammatory and anti-inflammatory responses, suggesting that it might act as a link between obesity and MS [22, 25]. Consequently, the proinflammatory condition in obesity most likely amplifies the immune response and thereby increases the risk of developing autoimmune inflammatory disorders, such as MS.

Some case reports have identified individuals developing symptoms of MS shortly after immense weight loss following bariatric surgery [26–28]. Conversely, anti-obesity medications based on glucagon-like peptide-1 receptor agonists appear to have a protective effect against the development of MS [29]. This suggests that weigh loss itself may not be a direct contributor to MS. In the present study, modest weigh loss after lifestyle behavioral treatment was not associated with increased risk of MS several year later. Over half of those who developed MS in the present study had class II obesity at their last recorded treatment visit, making direct comparisons to previously published reports challenging. Nevertheless, since obesity itself remains a significant factor contributing to MS risk, as demonstrated in this study, and is associated with MS progression and relapse [30, 31], prevention of high degree of obesity in childhood and adolescence is warranted.

Previous epidemiological research suggest that the association between early life high BMI and MS only is present for adolescent obesity and not childhood obesity [15–17]. However, studies using Mendelian randomization with estimated childhood BMI have suggested an effect of childhood BMI on subsequent MS risk [13, 14]. The current study extends these findings by using clinically obtained anthropometric measured in childhood. Consequently, the association between obesity and elevated risk of MS is shown to not be limited to adolescence but extends to childhood. Since treatment for pediatric obesity rarely results in obesity remission [19], it is plausible that early exposure to obesity leads to longer obesity duration. This may lead to a long-term pro-inflammatory response which could act as a catalyst, activating genes that are associated with the development of MS.

Parental MS was more common in the pediatric obesity cohort, which might give rise to a hypothesis of shared predisposition for MS and early obesity onset in the offspring. However, our study design prevents us from drawing any conclusions about causality. A genetic study, using Mendelian randomization, found little evidence of genetic pleiotropy between genes associated with high BMI in childhood and MS later in life [13], which contradicts a shared genetic predisposition. Another plausible explanation for this observation could be the strong hereditary nature of obesity. Consequently, parental BMI may be higher in the obesity cohort, potentially elevating the prevalence of parental MS. Future research should explore the potential synergistic relationship between MS heredity and childhood obesity in increasing MS risk. This research is vital for unraveling the intricate genetic and environmental interactions contributing to MS and obesity.

Despite the large-scale longitudinal study with individual-level data, some limitations should be acknowledged. Firstly, there are several risk factors known to be associated with MS that were not possible to control for, such as smoking, Epstein-Barr viral infection, and low vitamin D levels [16]. Secondly, no genetic data was available, but we were able to adjust for parental MS. Thirdly, as BORIS only includes anthropometric data from clinical visits, obesity duration prior to treatment cannot be determined. Fourthly, obesity status in the control group was unknown, which could cause an underestimation of obesity as a risk factor for MS. Fifthly, the median age at follow-up was 20.8 years of age, which should be kept in mind since MS typically develops between 20 and 40 years of age. Yet, we could observe an effect of pediatric obesity on MS.

## Conclusions

Both childhood and adolescent obesity are associated with an increased risk of MS. Moreover, a dose-response relationship between the degree of obesity and the risk of future MS was indicated. Given that weight reduction decreases obesity-induced inflammation, a key factor believed to mediate the link between obesity and MS, our study emphasizes the importance of preventing high degree of obesity early in life.

## Supplementary information

Supplementary file

## Data Availability

Patient-level data cannot be shared publicly because of third-party data. Given that an ethical approval is obtained, any individual may apply for data from Statistics Sweden via information@scb.se, the Swedish National Board of Health and Welfare via registerservice@socialstyrelsen.se, and the Swedish Childhood Obesity Treatment Register via http://www.e-boris.se/in-english/.

## References

[1] Lindberg L, Danielsson P, Persson M, Marcus C, Hagman E. Association of childhood obesity with risk of early all-cause and cause-specific mortality: A Swedish prospective cohort study. PLoS Med. 2020;17:e1003078. 32187177 10.1371/journal.pmed.1003078 PMC7080224

[2] Marcus C, Danielsson P, Hagman E. Pediatric obesity-Long-term consequences and effect of weight loss. J Intern Med. 2022;292:870–91. 35883220 10.1111/joim.13547 PMC9805112

[3] Lindberg L, Hagman E, Danielsson P, Marcus C, Persson M. Anxiety and depression in children and adolescents with obesity: a nationwide study in Sweden. BMC Med. 2020;18:30. 32079538 10.1186/s12916-020-1498-z PMC7033939

[4] Lindberg L, Persson M, Danielsson P, Hagman E, Marcus C. Obesity in childhood, socioeconomic status, and completion of 12 or more school years: a prospective cohort study. BMJ Open. 2021;11:e040432. 33707266 10.1136/bmjopen-2020-040432 PMC7957136

[5] Munger KL, Bentzen J, Laursen B, Stenager E, Koch-Henriksen N, Sorensen TI, et al. Childhood body mass index and multiple sclerosis risk: a long-term cohort study. Mult Scler. 2013;19:1323–9. 23549432 10.1177/1352458513483889 PMC4418015

[6] Hedstrom AK, Olsson T, Alfredsson L. High body mass index before age 20 is associated with increased risk for multiple sclerosis in both men and women. Mult Scler. 2012;18:1334–6. 22328681 10.1177/1352458512436596

[7] Mandato C, Colucci A, Lanzillo R, Staiano A, Scarpato E, Schiavo L et al. Multiple Sclerosis-Related Dietary and Nutritional Issues: An Updated Scoping Review with a Focus on Pediatrics. Children. 2023;10:1022. 10.3390/children10061022 PMC10297186 37371254

[8] Pakpoor J, Schmierer K, Cuzick J, Giovannoni G, Dobson R. Estimated and projected burden of multiple sclerosis attributable to smoking and childhood and adolescent high body-mass index: a comparative risk assessment. Int J Epidemiol. 2021;49:2051–7. 32844186 10.1093/ije/dyaa151

[9] Milles P, De Filippo G, Maurey H, Tully T, Deiva K, KidBiosep. Obesity in Pediatric-Onset Multiple Sclerosis: A French Cohort Study. Neurol Neuroimmunol Neuroinflamm. 2021;8:e1044. 10.1212/NXI.0000000000001044 PMC8293287 34285094

[10] Marrodan M, Farez MF, Balbuena Aguirre ME, Correale J. Obesity and the risk of Multiple Sclerosis. The role of Leptin. Ann Clin Transl Neurol. 2021;8:406–24. 33369280 10.1002/acn3.51291 PMC7886048

[11] Hedstrom AK, Lima Bomfim I, Barcellos L, Gianfrancesco M, Schaefer C, Kockum I, et al. Interaction between adolescent obesity and HLA risk genes in the etiology of multiple sclerosis. Neurology. 2014;82:865–72. 24500647 10.1212/WNL.0000000000000203 PMC3959752

[12] Gianfrancesco MA, Acuna B, Shen L, Briggs FB, Quach H, Bellesis KH, et al. Obesity during childhood and adolescence increases susceptibility to multiple sclerosis after accounting for established genetic and environmental risk factors. Obes Res Clin Pr. 2014;8:e435–47. 10.1016/j.orcp.2014.01.002 PMC4180039 25263833

[13] Harroud A, Mitchell RE, Richardson TG, Morris JA, Forgetta V, Davey Smith G, et al. Childhood obesity and multiple sclerosis: A Mendelian randomization study. Mult Scler. 2021;27:2150–8. 33749377 10.1177/13524585211001781

[14] Jacobs BM, Noyce AJ, Giovannoni G, Dobson R. BMI and low vitamin D are causal factors for multiple sclerosis: A Mendelian Randomization study. Neurol Neuroimmunol Neuroinflamm. 2020;7:e662. 10.1212/NXI.0000000000000662 PMC6975169 31937597

[15] Hedstrom AK, Olsson T, Alfredsson L. Body mass index during adolescence, rather than childhood, is critical in determining MS risk. Mult Scler. 2016;22:878–83. 26362895 10.1177/1352458515603798

[16] Olsson T, Barcellos LF, Alfredsson L. Interactions between genetic, lifestyle and environmental risk factors for multiple sclerosis. Nat Rev Neurol. 2017;13:25–36. 27934854 10.1038/nrneurol.2016.187

[17] Munger KL, Chitnis T, Ascherio A. Body size and risk of MS in two cohorts of US women. Neurology. 2009;73:1543–50. 19901245 10.1212/WNL.0b013e3181c0d6e0 PMC2777074

[18] Cole TJ, Lobstein T. Extended international (IOTF) body mass index cut-offs for thinness, overweight and obesity. Pediatr Obes. 2012;7:284–94. 22715120 10.1111/j.2047-6310.2012.00064.x

[19] Hagman E, Danielsson P, Lindberg L, Marcus C, Committee BS. Paediatric obesity treatment during 14 years in Sweden: Lessons from the Swedish Childhood Obesity Treatment Register-BORIS. Pediatr Obes. 2020;15:e12626. 32074662 10.1111/ijpo.12626

[20] Ludvigsson JF, Almqvist C, Bonamy AK, Ljung R, Michaelsson K, Neovius M, et al. Registers of the Swedish total population and their use in medical research. Eur J Epidemiol. 2016;31:125–36. 26769609 10.1007/s10654-016-0117-y

[21] Murley C, Friberg E, Hillert J, Alexanderson K, Yang F. Validation of multiple sclerosis diagnoses in the Swedish National Patient Register. Eur J Epidemiol. 2019;34:1161–9. 31493189 10.1007/s10654-019-00558-7 PMC7010617

[22] Montgomery TL, Peipert D, Krementsov DN. Modulation of multiple sclerosis risk and pathogenesis by the gut microbiota: Complex interactions between host genetics, bacterial metabolism, and diet. Immunol Rev. 2024;325:131–51. 38717158 10.1111/imr.13343 PMC11338732

[23] Sawyer SM, Azzopardi PS, Wickremarathne D, Patton GC. The age of adolescence. Lancet Child Adolesc Health. 2018;2:223–8. 30169257 10.1016/S2352-4642(18)30022-1

[24] Ji Z, Wu S, Xu Y, Qi J, Su X, Shen L. Obesity Promotes EAE Through IL-6 and CCL-2-Mediated T Cells Infiltration. Front Immunol. 2019;10:1881. 31507583 10.3389/fimmu.2019.01881 PMC6718738

[25] Samara A, Cantoni C, Piccio L, Cross AH, Chahin S. Obesity, gut microbiota, and multiple sclerosis: Unraveling the connection. Mult Scler Relat Disord. 2023;76:104768. 37269641 10.1016/j.msard.2023.104768

[26] Alanazy MH, Alomar MA, Aljafen BN, Muayqil TA. Multiple sclerosis and myasthenia gravis following severe weight loss. Neuroscience. 2018;23:158–61. 10.17712/nsj.2018.2.20170343 PMC8015450 29664459

[27] Asakly S, Magen-Rimon R, Ighbariya A, Marjih-Shallufi M, Ben-Porat T, Ravid S, et al. Bariatric Surgery-Associated Myelopathy. Obes Facts. 2021;14:431–9. 34311464 10.1159/000515374 PMC8406245

[28] Bitarafan S, Amani K, Sahraian MA, Sarraf P, Soltani D, Moghadasi AN, et al. The first attack of multiple sclerosis presented immediately after voluntary and intensive weight loss: A case series. Iran J Neurol. 2017;16:41–42. 28717433 PMC5506755

[29] Shirani A, Cross AH, Stuve O. Exploring the association between weight loss-inducing medications and multiple sclerosis: insights from the FDA adverse event reporting system database. Ther Adv Neurol Disord. 2024;17:17562864241241383. 38566910 10.1177/17562864241241383 PMC10986166

[30] Marrie RA. Comorbidity in multiple sclerosis: implications for patient care. Nat Rev Neurol. 2017;13:375–82. 28303911 10.1038/nrneurol.2017.33

[31] Tettey P, Simpson S, Taylor B, Ponsonby AL, Lucas RM, Dwyer T, et al. An adverse lipid profile and increased levels of adiposity significantly predict clinical course after a first demyelinating event. J Neurol Neurosurg Psychiatry. 2017;88:395–401. 28320766 10.1136/jnnp-2016-315037

