# Supplementary material for: Pediatric obesity and the risk of multiple sclerosis: a nationwide prospective cohort study
Source: Int J Obes (Lond). 2025 Jan 30;49(6):1031–6. doi: 10.1038/s41366-025-01727-3 (PMC12158760; doi:10.1038/s41366-025-01727-3)
Supplement: Supplementary file 1 — Supplementary file [file 41366_2025_1727_MOESM1_ESM.docx]

# Supplementary material for

**Pediatric obesity and the risk of multiple sclerosis: A nationwide prospective cohort study**

Hagman E, Putri RR, Danielsson P, Marcus C

| Supplementary Table 1. ICD-10 codes used at exclusion | |
| --- | --- |
| Diagnosis/Procedure | ICD-10 code |
| Down syndrome | Q90 |
| Prader-Willi syndrome | Q871 |
| Laurence-Moon-Biedi syndrome | Q878 |
| Russel-Silver syndrome | Q871G |
| Noonan syndrome | Q871E |
| Klinefelter’s syndrome | Q98 |
| Fragile X chromosome | Q992 |
| Turner syndrom | Q96 |
| Chraniopharyngioma | D353, D444, C752 |
